# Coinfection with malaria and intestinal parasites, and its association with anaemia in children in Cameroon

**DOI:** 10.1186/s40249-015-0078-5

**Published:** 2015-10-06

**Authors:** Anna Longdoh Njunda, Shuri Ghasarah Fon, Jules Clement Nguedia Assob, Dickson Shey Nsagha, Tayong Dizzle Bita Kwenti, Tebit Emmanuel Kwenti

**Affiliations:** Department of Medical Laboratory Sciences, University of Buea, P.B. 63, Buea, Cameroon; Department of Public Health and Hygiene, University of Buea, P.B. 63, Buea, Cameroon; Department of Microbiology and Parasitology, University of Buea, P.B. 63, Buea, Cameroon

**Keywords:** Malaria, Intestinal parasites, Intestinal parasitic infections, Coinfection, Anaemia, Children, Prevalence, Cameroon

## Abstract

**Background:**

The purpose of this study was to determine the prevalence of coinfection with malaria and intestinal parasites, as well as to determine its association with anaemia in children aged 10 years and below in Muyuka, Cameroon.

**Materials and methods:**

This was a cross-sectional study. Participants were febrile children who were admitted to the Muyuka district hospital between April and October 2012. Blood and stool samples were collected from those participants who gave consent to take part in the study. Haemoglobin concentration (Hb) and complete blood count (CBC) were performed using an automated haematology analyser (Mindray®, BC-2800). Giemsa-stained blood film was examined to detect malaria parasites, while the formol-ether concentration technique was used to detect intestinal parasitic infections (IPIs). The Pearson’s chi-square, Student’s T-test and correlation analysis were all performed as part of the statistical analyses.

**Results:**

Four hundred and eleven (411) children successfully took part in this study. The prevalence of malaria, IPIs, malaria and IPI coinfection, and anaemia observed were 98.5 %, 11.9 %, 11.9 % and 44.8 %, respectively. Anaemia and IPIs were significantly associated with age; anaemia was more prevalent in children under five years of age (*p* = 0.000), whereas IPIs were more prevalent in children aged between five and 10 years (*p* = 0.006). The parasite species isolated included *Ascaris lumbricoides* (36 [73.5 %]), *Entamoeba histolytica/dispar* (9 [18.4 %]) and hookworm (4 [8.2 %]). The mean Hb observed was 10.64 g/dl (±1.82). A significant negative correlation was observed between malaria parasite density and Hb. There was no significant difference in the prevalence of anaemia among children infected with malaria, IPIs, or malaria and IPI coinfection, or among non-infected children. Similarly, the mean Hb did not differ among infected and non-infected children.

**Conclusion:**

This study showed that malaria and IPIs still constitute a major public health problem in the study area despite a lack of any significant association between these infections and anaemia. The findings suggest that there is a need for the implementation of control measures to curb the rate of malaria and IPIs in the study area.

## Background

Malaria and intestinal parasitic infections (IPIs) are among the most prevalent diseases in Sub-Saharan Africa (SSA). Although there has been a decline in the global prevalence of malaria (due to an increased number of funding bodies that have been contributing to the fight against the disease in the last decade), hundreds of thousands of people still die from the disease every year. The most vulnerable group is children [1]. In 2013, 198 million cases of malaria and 584,000 deaths were reported [2]. The majority of deaths from malaria occurred in children under 15 years of age in SSA [3]. Five protozoan species, namely *Plasmodium vivax*, *P. ovale*, *P. malariae*, *P. knowlesi* and *P. falciparum*, cause malaria, with the latter being the most virulent and accounting for the majority of malaria deaths [4, 5].

In the tropics, IPIs constitute a major public health problem, as these areas are often characterised by all the conditions favouring transmission of these infections, including a humid climate, unsanitary environments and poor socio-economic conditions. Helminths or protozoa, or both, cause IPIs. Neglected IPIs, particularly infection with helminths (helminthiasis), are a major cause of morbidity, especially in resource-limited settings [6]. The incidence of IPIs is approximately 50 % in developed countries and reaches up to 95 % in developing countries, with SSA having the highest burden of these infections [7, 8].

Due to malaria’s and IPI’s overlapping distribution, concomitant infection with malaria and intestinal parasites is common in developing countries, especially in SSA. Coinfection causes varying effects in the host. Concomitant infections in children have been shown to adversely affect their development and learning capabilities [9–11], and have been associated with increased susceptibility to other infections [12–14]. Studies have shown that individuals coinfected with more than one parasite species are at risk of increased morbidity [15–19], as well as an increased risk of developing more frequent and severe diseases due to interactions among infecting parasite species [12, 13, 20]. Concomitant infection with malaria and intestinal parasites is also associated with anaemia. Intestinal parasitic infections, especially those with hookworm and *Trichuris trichiura*, cause anaemia by increasing blood and iron loss in the intestinal tract. Meanwhile, malaria is associated with a decrease in the amount of haemoglobin, increased destruction of parasitised red blood cells (RBCs), shortened lifespan of non-parasitised RBCs and decreased production of RBCs in the bone marrow, which eventually leads to anaemia [21, 22].

Little research has been done to investigate the association between malaria and IPI coinfection with anaemia in children. Moreover, no study of this sort has ever been conducted in the health district of Muyuka, Southwest Region of Cameroon, an area characterised by high malaria transmission. Therefore, the present study was undertaken to determine the prevalence of malaria and IPI coinfection, as well as to determine its association with anaemia in children aged 10 years and below. The findings of this study can be used to inform intervention strategies.

## Methods

### Study design and duration

This was a cross-sectional study conducted between April and October 2012. The study participants were febrile children who were admitted to the Muyuka district hospital during the study period.

### Study area

Muyuka (4°43’18”N, 9°38’27”E) is a small town in the Fako Division, Southwest Region of Cameroon. It is situated some 31 km from Buea, the region’s capital. It is an administrative headquarter and is also a health district with one district hospital. There are two main seasons (the rainy season, which lasts from mid-March to October, and the dry season, which lasts from November to early March). The mean temperature ranges from 23 °C in the coldest months to 33 °C in the hottest months. The population is cosmopolitan, with about 118,470 inhabitants. The major ethnic group is the Balong tribe, followed by the Bakweri (however, this tribe do interact with smaller villages such as Ekata, Bafia, Yoke, Malende, Muyenge and Meanja). The main activity is farming, with cocoa being the major cash crop. The planning of this site has been poorly done, with settlements and several farms clustered around. There are breeding sites for *Anopheles* mosquitoes around homes. Hospital records show that malaria transmission occurs all year round, with peaks at the beginning of the rainy season (April and May).

### Study population

Eligible participants were children aged 10 years and below who resided in the Muyuka health district (from Ekata, Bafia, Yoke, Malende, Muyenge or Meanja), and were not on any antimalarial or antiparasitic drugs for at least two weeks prior to the study commencing.

### Specimen collection and processing

Once participants gave signed informed consent, their stool and blood samples were collected. Parents or guardians were instructed to put a teaspoon of stool into sterile leak-proof wide-neck stool containers. About 4 ml of whole blood was collected into EDTA anticoagulated tubes to perform the complete blood count (CBC). Thick and thin blood films were prepared for malaria microscopy.

### Determining the haemoglobin concentration (Hb)

The CBC was performed using the Mindray® automatic haematology analyser (BC-2800, Shenzhen Mindray Bio-Medical Electronics Co., Ltd, Shenzhen, P.R. China). Haemoglobin concentration (Hb) was obtained from the CBC results. The levels of anaemia were defined as stipulated by the World Health Organization (WHO) [23]: children under five years of age, Hb < 11 g/dl; and children aged five to 10 years, Hb < 11.5 g/dl. Further classification was done to determine severe, moderate and mild anaemia cases, which produced values of <6 g/dl, 6.1–8 g/dl and 8.1–10.9 g/dl, respectively [24].

### Parasitological analysis

1. *Detection of malaria parasites*: Thick and thin blood films were prepared and stained with 10 % Giemsa and examined using methods previously described [25]. If parasites were observed, the density was then determined by counting the number of parasites against 200 leucocytes. The parasite density was obtained by dividing the number of parasites by 200 and multiplying the result by the actual white blood cell count of the patient [26].
2. *Stool processing and detection of intestinal parasites using the formol-ether concentration technique:* Using an applicator stick, about one gram of stool was emulsified in about 7 ml of 10 % formol water in a screw-cap tube. This process has previously been described by Cheesbrough [27].

### Statistical analysis

Data collected were entered into an Excel spreadsheet and analysed using the Stata® version 12.1 software (StataCorp LP, Texas, USA). The statistical tests performed included the Pearson’s Chi-square for the group comparison, the Student’s T-test to compare group means, and correlation analysis to determine the association between parasite density and Hb. Statistical significance was set at *p* < 0.05.

### Ethical considerations

The present study was approved by the institutional review board of the Faculty of Health Sciences, University of Buea, Cameroon. Administrative clearance was obtained from the delegation of public health in the Southwest Region of Cameroon. Participation was voluntary and the objectives of the study were explained to all the participants (parents of children). Parents or guardians signed consent forms on behalf of their children.

## Results

Four hundred and fifty-three (453) children were approached to participate, with 411 (90.7 %) being successful and consequently providing stool samples and blood specimens. The mean (±SD) age of the participants was 41.3 (±33.53) months. There were 214 (52.07 %) females and 197 (47.93 %) males.

Four hundred and five (405) participants were positive for malaria, resulting in a prevalence of 98.5 % (95 % CI: 97.4–99.7). The prevalence was higher among males (196/197 [99.5 %]) than females (209/214 [97.7 %]), however, no significant association was observed between the prevalence of malaria and gender (χ^2^ = 2.385, *p* = 0.123). The prevalence of malaria was higher in children aged between five to 10 years (115/116 [99.1 %]) compared to children below five years of age (290/295 [98.3 %]). Again, no significant difference was observed between the prevalence of malaria and age (χ^2^ = 0.4015, *p* = 0.526). The parasite density ranged between 65 and 160,523 (mean ± SD = 10,332.67 ± 24,746.6).

Among the 411 participants, 49 were positive for intestinal parasites, resulting in a prevalence of 11.9 % (95 % CI: 8.78–15.1). Infections with helminths were more common than those with protozoa (81.6 % vs. 18.4 %). As well as that, IPIs were more prevalent (χ^2^ = 7.64, *p* = 0.006) in children aged between five and 10 years (22/116 [19.0 %]) compared to children below five years of age (27/295 [9.2 %]). The prevalence of infection was higher in males (27/197 [13.7 %]) than in females (22/214 [10.3 %]), however, no significant association was observed between the prevalence of IPIs and gender (χ^2^ = 1.146, *p* = 0.284). Infection with the *Ascaris lumbricoides* species was the most common IPI identified (36 [73.5 %]), followed by *Entamoeba histolytica/dispar* (9 [18.4 %]) and hookworm (4 [8.2 %]). No significant association was observed between the prevalence of parasite species and age (χ^2^ = 0.6072, *p* = 0.738). Infection with more than one species of intestinal parasites (polyparasitism) was not observed in this study.

All children infected with intestinal parasites were also coinfected with malaria. This means that the prevalence of malaria and IPI coinfection is also 11.9 %.

In this study, 184 of the 411 participants were anaemic, resulting in a prevalence of 44.8 % (95 % CI: 39.9–49.7). The prevalence rates of mild, moderate and severe anaemia were 69.6 % (128), 17.4 % (32) and 13.0 % (24), respectively. Anaemia was more prevalent (χ^2^ = 17.41, *p* = 0.000) in children aged five years or below (151/295 [51.2 %]) compared to children between five and 10 years of age (33/116 [28.5 %]). The prevalence of anaemia was higher in females (101/214 [47.2 %]) compared to males (83/197 [42.1 %]), however, no significant association was observed between prevalence of anaemia and gender (χ^2^ = 1.064, *p* = 0.302).

Out of the participants who were positive for malaria, 183 (45.2 %) were anaemic, however, no significant association was observed between the prevalence of malaria and anaemia (χ^2^ = 1.95, *p* = 0.163). Among the participants with IPIs, 27 (55.1 %) were anaemic. Again, no significant association was observed between the prevalence of IPIs and anaemia (χ^2^ = 2.4, *p* = 0.121). Species-specific analysis didn’t reveal a significant association between the different species of intestinal parasites and anaemia (χ^2^ = 0.17, *p* = 0.920) (see Table 1).

**Table 1:** Association of anaemia, malaria and IPIs in the study population

Type of infection	N	Anaemia		Chi-square	*p*-value
		Absent (%)	Present (%)		
Malaria	405	222 (54.8)	183 (45.2)	χ^2^ = 1.95	0.163
IPIs*	49	22 (44.9)	27 (55.1)	χ^2^ = 2.4	0.121
Parasite species					
Hookworm	4	2 (50)	2 (50)		
* Ascaris lumbricoides*	36	17 (47.2)	19 (52.8)	χ^2^ = 0.17	0.920
* Entamoeba histolytica/dispar*	9	3 (33.3)	6 (66.7)		

*All patients with IPIs were also infected with malaria, which implies coinfection

The prevalence of severe anaemia was 13.1 %, 14.8 % and 14.8 % among the participants with malaria, IPIs, and malaria and IPI coinfection, respectively (see Table 2). No significant difference was observed between the degree of anaemia and type of infection (χ^2^ = 0.277, *p* = 0.992).

**Table 2:** Association of different levels of anaemia, and malaria and IPIs in the study population

Type of infection	n	Type of anaemia		
		Mild (%)	Moderate (%)	Severe (%)
Malaria	183	127 (69.4)	32 (17.5)	24 (13.1)
IPIs*	27	19 (70.4)	4 (14.8)	4 (14.8)
Total	210	146 (69.5)	36 (17.1)	28 (13.3)

*All patients with IPIs were also infected with malaria, which implies coinfection

**Fig. 1:**
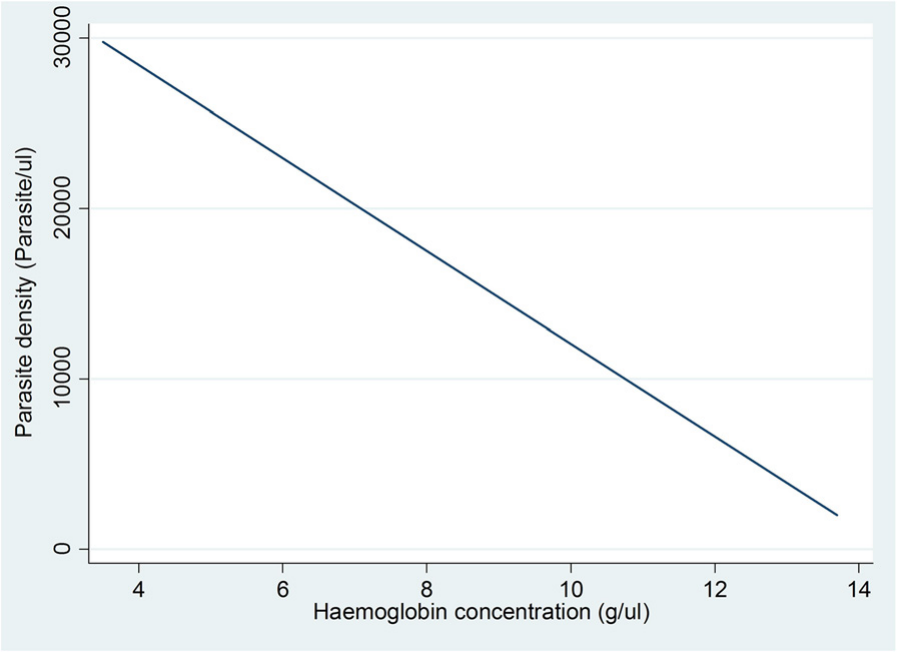
Plot of Hb against malaria parasite density. The figure shows an inverse relationship between malaria parasite density and Hb

In this study, the mean (±SD) Hb was 10.64 g/dl (±1.82) (range: 3.5–13.7). The mean Hb did not differ significantly among children who were infected with malaria and those who were not (*p* = 0.127), or between children with IPIs and those without (*p* = 0.14) (see Table 3). However, a significant negative correlation was observed between Hb and malaria parasite density (r =–0.23, *p* = 0.000) (see Fig. 1).

**Table 3:** Comparison of Hb (g/dl) among the study population

Type of infection		Mean Hb±SD	*p*-values
Malaria	Negative	11.48±1.47	0.127
	Positive	10.63±1.82	
IPIs*	Negative	10.68±1.8	0.14
	Positive	10.38±1.98	

*All patients with IPIs were also infected with malaria, which implies coinfection

## Discussion

In the present study, the prevalence of malaria among the study participants was 98.5 %, which is very high compared to the prevalence reported in children in other areas of the Southwest Region of Cameroon. This includes the 50.7 % reported in villages in Mbonge [11], 33.8 % in Limbe and Buea [28], and 40.6 % in the Centre Region of Cameroon [29]. These discrepancies could be explained by the fact that the present study was hospital-based in which febrile children were enrolled, compared to the other studies in which apparently healthy children either from schools or the community were enrolled. Compared to similar studies done in other countries, the prevalence of malaria we found was also very high; 28.8 % was reported in Southern Ethiopia [30], 29.8 % in Tanzania [19] and 11.5 % in northwest Ethiopia [31]. These differences could be attributed to the different levels of malaria endemicity in these areas compared to our study, which was concerned with holoendemic and hyperendemic malaria. Furthermore, our study was conducted during the rainy season, when malaria transmission reaches its peak.

No significant associations were observed between the prevalence of malaria and age in this study, which is in line with several studies done elsewhere [19, 29], but contrary to the studies done by Degarege et al. [30] and Alemu et al. [31]. Similarly, malaria was not observed to be associated with gender, which conforms to studies conducted elsewhere [19, 29, 30]. However, in the study conducted by Alemu et al. [31], the prevalence of malaria was observed to be higher in males than in females.

In the present study, the prevalence of IPIs was 11.9 %, which is lower compared to the 34.7 % reported in communities around Dschang, in the West Region of Cameroon [32], 22.7 % in Thailand [33] and 34.2 % in Ethiopia [34]. These discrepancies could be attributed to the differences in diagnostic techniques used to detect parasites, as well as geographical differences. Regular deworming campaigns, run by the Cameroon’s Ministry of Public Health, have taken place in the study area, which may account for the lower prevalence of IPIs. The prevalence of helminths was higher than the prevalence of protozoa (81.6 vs. 18.4 %), which is in consonance with some studies [32, 34], but in contrast with others [35, 36]. Intestinal parasitic infections were more prevalent in children aged five years and above (*p* = 0.006), which conforms to studies done elsewhere [29, 32]. This could be attributed to differences in exposure levels in children as they grow. The intestinal parasites isolated in this study were *Ascaris lumbricoides* (73.5 %), *Entamoeba histolytica/dispar* (18.4 %) and hookworm (8.2 %). *Ascaris lumbricoides* species being the predominant parasite species causing infection in children is in line with other studies conducted in other areas of Cameroon [11, 29, 37, 38], and elsewhere [39]. Infection with more than one intestinal parasite (polyparasitism) was not observed in this study. The intensity of isolates was not determined because the Kato-Katz technique was not employed (due to problems with logistics), and this constituted a major limitation. Furthermore, we were unable to separate *E. histolytica* from *E. dispar*.

All the children with IPIs in the present study also had malaria, resulting in a prevalence of 11.9 % for coinfection. This figure is low compared to the 26.1 % reported by Makoge et al. [11] and 60 % reported in Tanzania [19], but high compared to the 7.7 % reported in Southwest Ethiopia [40]. The low prevalence of malaria and IPI coinfection could be attributed to the same factors responsible for the low prevalence of IPIs in general, as outlined above.

The prevalence of anaemia was 44.8 % in this study. This is high and may have consequences on learning and development of children if not addressed rapidly. The prevalence of anaemia observed in this study is lower compared to the 57.6 % reported in Mbonge [11], but higher compared to the 33.5 % reported in the Centre Region of Cameroon [29], 19.8 % in Limbe and Buea in the Southwest Region of Cameroon [28], and 10.9 % in Ethiopia [31]. Again, this could be due to the differences in study design. Anaemia was more significantly more prevalent in children below five years of age (*p* = 0.000), but no significant association was observed between the prevalence of anaemia and gender. Young children are more vulnerable to anaemia, a situation complicated by the presence of infections with bacteria, malaria and intestinal parasites. The association between anaemia and age aligns with some studies [11, 17], but is contrary to a study conducted by Alemu et al. [31], in which no association between the prevalence of anaemia and age was observed. No association was found between the prevalence of anaemia and gender in studies conducted elsewhere [11, 29, 31] either. In this study, 13.3 % of participants had severe anaemia, which is above the range of 1.3–6.4 % estimated for severe anaemia in children residing in malaria-endemic areas of Africa [41]. No significant association was observed between anaemia and malaria monoinfection, IPI monoinfection, and malaria and IPI coinfection. No significant association between infection and the degree of anaemia was observed. Makoge et al. [11] also found no association between malaria or IPIs and anaemia, and Tsuyuoka et al. [37] likewise did not observe any association between intestinal parasites and anaemia. However, other studies conducted elsewhere have reported a significant association between malaria, intestinal helminths and anaemia [19, 31, 42–44]. Malaria is the most important infectious cause of anaemia, especially in developing countries [45]. Not observing any significant association between malaria and anaemia could be attributed to the very high prevalence of malaria in this study; only six children were not infected with malaria. Nevertheless, there are other important causes of anaemia including malnutrition, which is rampant among impoverished communities in developing countries [46].

The association between intestinal parasites, especially hookworm, and the development of anaemia is well known, however, infection with other parasites such as *Entamoeba histolytica* can also lead to anaemia through blood loss in diarrhoea. In this study, species-specific analysis didn’t reveal a significant association between the different parasite species and anaemia. This differs from the study conducted by Osazuwa et al. [38], in which a significant association was observed between hookworm and *Ascaris lumbricoides*, and anaemia.

The mean (±SD) Hb of participants in this study was 10.64±1.82. No significant difference in the mean Hb was observed between malaria infected and non-malaria infected children (*p* = 0.127), or between participants infected with intestinal parasites and those not infected (*p* = 0.14). The lack of a significant association between malaria and Hb could be attributed to the very high prevalence of malaria in this study. In contrast, a study conducted by Yentür et al. [44] observed that IPIs significantly lowered Hb in infected children, but this depended on the intensity of the infection.

A significant negative correlation between malaria parasite density and Hb was observed in this study (r =–0.23, *p* = 0.000). This conforms to a study done in Nigeria by Achidi et al. [47]. This is because when the parasite density increases, there’s an increasing destruction of RBCs and an eventual decrease in the Hb. This evaluation was not feasible with IPIs because the parasite intensity was not determined. In another study, Cornet et al. [48] did not observe such a correlation, although they did identify malaria as a risk factor for anaemia.

## Conclusion

This study revealed a very high prevalence of malaria and anaemia, and a lower prevalence of IPIs, in the study area. *Ascaris lumbricoides*, *Entamoeba histolytica/dispar* and hookworm all caused IPIs in the study population. No significant association was observed between malaria, IPIs, or malaria and IPI coinfection with anaemia, but a significant negative correlation was observed between malaria parasite density and Hb. These findings therefore underline the need for intervention programmes to work at reducing the disease burden in the study area. Measures including education on personal hygiene and environmental sanitation, regular use of chemotherapy, and use of antimalarial (intermittent preventive treatment) and anthelminthic medication should be encouraged.

## Abbreviations

CBC: Complete blood count
Hb: Haemoglobin concentration
IPI: Intestinal parasitic infection
RBC: Red blood cell
SSA: Sub-Saharan Africa
WHO: World Health Organization
